# Arithmetic hip-knee-ankle angle and stressed hip-knee-ankle angle: equivalent methods for estimating constitutional lower limb alignment in kinematically aligned total knee arthroplasty

**DOI:** 10.1007/s00167-022-07038-8

**Published:** 2022-07-11

**Authors:** Payam Tarassoli, Jil A. Wood, Darren B. Chen, Will Griffiths-Jones, Johan Bellemans, Samuel J. MacDessi

**Affiliations:** 1 CPAK Research Group, Sydney, Australia; 2 Sydney Knee Specialists, Suite 201, Level 2, 131 Princes Hwy, Kogarah, NSW 2217 Australia; 3 North Devon District Hospital, Raleigh Heights, Barnstaple, UK; 4 ZOL Hospitals, Genk, Belgium; 5 ArthroClinic, Leuven, Belgium; 6 St George and Sutherland Clinical School, University of New South Wales, Sydney, NSW Australia

**Keywords:** Constitutional alignment, Arithmetic HKA, Stressed HKA, Kinematic alignment, Total knee arthroplasty

## Abstract

**Purpose:**

Kinematically aligned total knee arthroplasty (KA TKA) relies on precise determination of constitutional alignment to set resection targets. The arithmetic hip-knee-ankle angle (aHKA) is a radiographic method to estimate constitutional alignment following onset of arthritis. Intraoperatively, constitutional alignment may also be approximated using navigation-based angular measurements of deformity correction, termed the stressed HKA (sHKA). This study aimed to investigate the relationship between these methods of estimating constitutional alignment to better understand their utility in KA TKA.

**Methods:**

A radiological and intraoperative computer-assisted navigation study was undertaken comparing measurements of the aHKA using radiographs and computed tomography (CT-aHKA) to the sHKA in 88 TKAs meeting the inclusion criteria. The primary outcome was the difference in the paired means between the three methods to determine constitutional alignment (aHKA, CT-aHKA, sHKA). Secondary outcomes included testing agreement across measurements using Bland-Altman plots and analysis of subgroup differences based on different patterns of compartmental arthritis.

**Results:**

There were no statistically significant differences between any paired comparison or across groups (aHKA vs. sHKA: 0.1°, *p* = 0.817; aHKA vs. CT-aHKA: 0.3°, *p* = 0.643; CT-aHKA vs. sHKA: 0.2°, *p* = 0.722; ANOVA, *p* = 0.845). Bland-Altman plots were consistent with good agreement for all comparisons, with approximately 95% of values within limits of agreement. There was no difference in the three paired comparisons (aHKA, CT-aHKA, and sHKA) for knees with medial compartment arthritis. However, these findings were not replicated in knees with lateral compartment arthritis.

**Conclusions:**

There was no significant difference between the arithmetic HKA (whether obtained using CT or radiographs) and the stressed HKA in this analysis. These findings further validate the preoperative arithmetic method and support use of the intraoperative stressed HKA as techniques to restore constitutional lower limb alignment in KA TKA.

**Level of evidence:**

III.

## Introduction

Recent strategies in the pursuit of more favourable outcomes following total knee arthroplasty (TKA) have focused on restoration of constitutional lower limb alignment and joint line obliquity. Termed kinematic alignment (KA), this method has been shown to more reliably restore soft tissue laxities and native joint kinematics [6, 28–30, 43, 54]. However, with the progressive deformity that follows loss of articular cartilage, determination of constitutional lower limb alignment is challenging [10].

The recently described arithmetic hip–knee–ankle angle (aHKA) uses preoperative radiographs to estimate constitutional alignment following the onset of arthritis by measurement of angles unaffected by joint space narrowing, validated to apply to both arthritic and non-arthritic populations [18] and in comparison with contralateral normal limbs [33]. Investigating an arthritic population, McEwen et al. demonstrated that constitutional alignment can also be approximated intraoperatively during computer-assisted TKA by stressing the collateral ligaments to reverse the direction of arthritic deformity, thereby producing a “stressed” HKA (sHKA) [38].

This technique can then be used to set distal femoral and proximal tibial resections to restore each patient’s unique limb alignment [31, 38, 39]. Although preoperative stress radiographs have demonstrated utility in defining the constitutional alignment and need for soft tissue releases intraoperatively [20, 27, 46], it is unknown whether the intraoperative sHKA method correlates with the aHKA. Further, it is unknown if the sHKA is similarly predictive of the constitutional alignment based on whether the deformity has resulted from medial or lateral compartment OA. As both the aHKA and sHKA are methods that negate the contribution of joint space narrowing in osteoarthritis, it follows that they would yield equivalent values in direct comparison. Furthermore, although reasonable correlation has been shown between radiographs and computed tomography (CT) in coronal plane assessment of knee alignment [3, 16, 23, 50, 52], the derivation of the aHKA has yet to be applied to CT imaging.

The purpose of this study was to determine if the preoperative aHKA and the intraoperative sHKA are related, thereby validating the reliability of the sHKA to act as a surrogate target for constitutional alignment, and whether this comparison is dependent on the compartmental pattern of OA. Additionally, we wanted to investigate whether CT-derived aHKA (CT-aHKA), measured in preoperative planning for robotic TKA [11], would be equivalent to the aHKA calculated from radiographs and then to consider if the same relationship exists between the CT-aHKA and sHKA. The primary hypothesis was that in patients undergoing primary TKA for osteoarthritis (OA), the aHKA, sHKA, and CT-aHKA would not be significantly different in the same knee. The secondary hypothesis was that in the same cohort of patients, there would be statistical agreement between measurements of aHKA, sHKA and CT-aHKA in the same knee. Identifying a direct relationship between the sHKA and aHKA would further confirm reliability and lend support to routine use in restoring constitutional alignment in KA TKA.

## Methods

### Study design

A retrospective study was undertaken to compare measurements of the arithmetic HKA, using weight-bearing long-leg radiographs for the aHKA, computed tomography for CT-aHKA, and intraoperative measurements for the stressed HKA (sHKA). Ethics approval was granted from the Hunter New England Local Health District Human Research Ethics Committee, #EX201905-02. All investigations and procedures undertaken were in accordance with the ethical standards of the institutional research committee and with the 1964 Declaration of Helsinki and its later amendments or comparable ethical standards.

### Study group

**Fig. 1:**
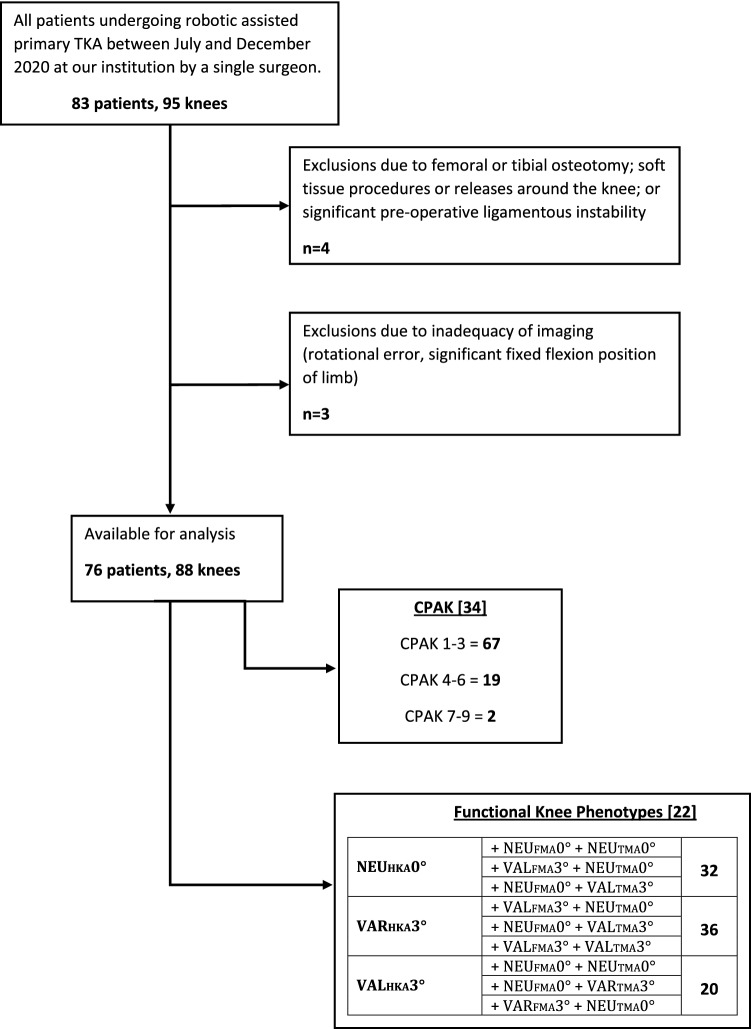
Study flow chart indicating patient inclusion/exclusion and knee phenotypes of the study group

Radiographic and intraoperative data were collected from a consecutive series of patients who underwent robotic-assisted primary TKA (Mako Triathlon, Stryker, Kalamazoo, MI, USA), for end-stage degenerative OA between July and December 2020. All patients who were included had at minimum unicompartmental knee OA with grade 3 or 4 changes as per Kellgren-Lawrence [25]. Following screening for imaging adequacy and exclusion criteria, 88 radiographs with corresponding intraoperative data were available for analysis in 76 patients. Surgeries were performed by a single surgeon at a private hospital in Sydney, Australia. The flow chart in Fig. 1 illustrates inclusion/exclusion criteria and a summary of the broad knee phenotypes [22, 34] of the study group.

### Radiographic technique

Weight-bearing long-leg radiographs in the “stand-at-attention” position were taken using the technique described by Paley [44]. All radiographs were screened for inclusion by an orthopaedic fellow based on deviation from accepted rotational alignment; those of inadequate quality were excluded. Inclusion criteria were defined by the patellae being positioned symmetrically facing forward, the lesser trochanters having a similar shape, and the proximal tibiofibular joints having similar overlap. In addition, significant fixed flexion deformity of the arthritic limb was assessed by noting asymmetry of the intercondylar outline.

CT-derived values for the aHKA were calculated from coronal plane geometry reported within software for the Mako robotic system. This software utilises preoperative CT imaging, using a pre-defined protocol to maximise bony architecture [11, 50]. The DICOM images were transferred to the system software for determination of lower limb anatomical landmarks by the Mako product specialist.

### Radiographic analysis for aHKA

**Fig. 2:**
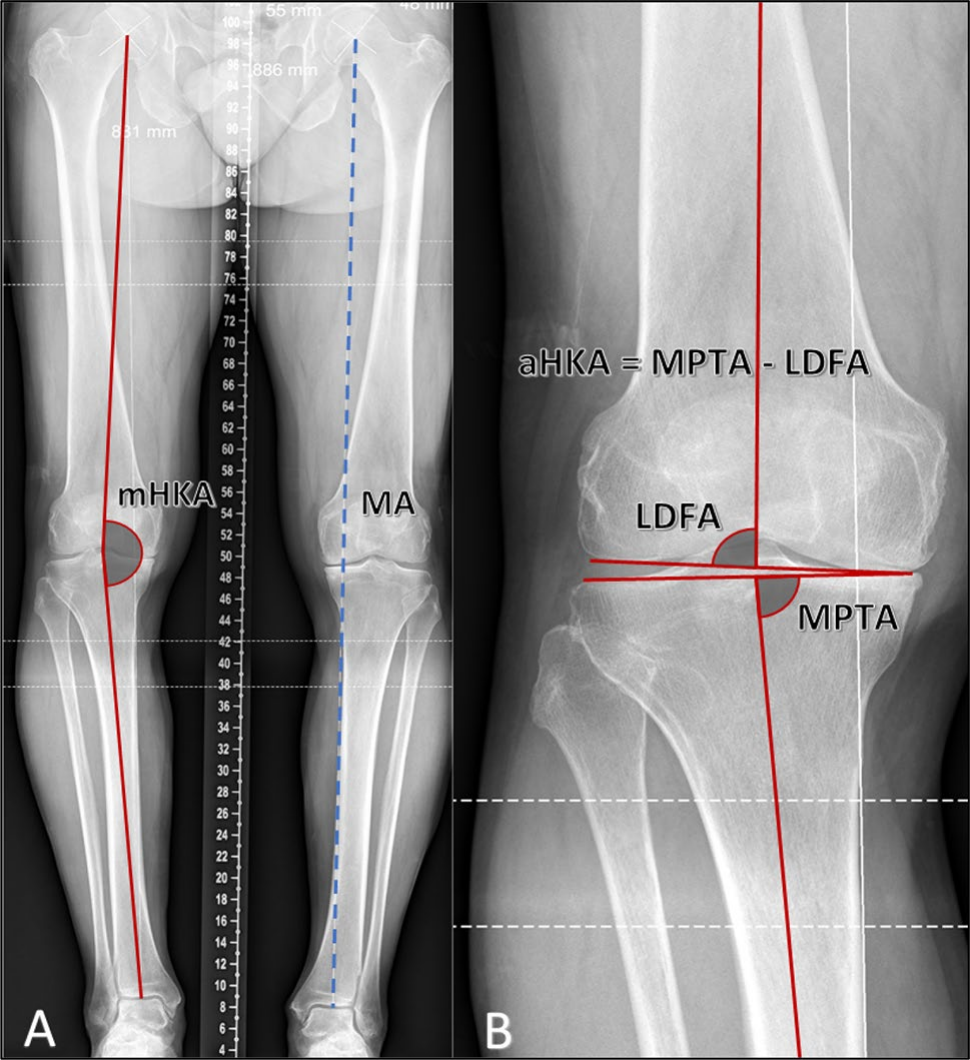
Determination of the arithmetic HKA angle on radiographs. **A** Full preoperative long leg standing radiograph. In the right limb, the mechanical HKA (mHKA) is highlighted. The mHKA is the angle subtended by the MA femur and the MA tibia. The MA of the lower limb, shown on the left leg, is the line marked from the centre of the hip joint to the centre of the ankle joint. **B** Calculation of constitutional alignment in the arthritic knee using the aHKA algorithm, which subtracts the lateral distal femoral angle (LDFA) from the medial proximal tibial angle (MPTA). *mHKA* mechanical hip-knee-ankle angle, *MA* mechanical axis, *aHKA* arithmetic hip-knee-ankle angle, *LDFA* lateral distal femoral angle, *MPTA* medial proximal tibial angle

The mechanical axis (MA) of the femur was defined as a line from the centre of the femoral head to the centre of the distal femur at the knee joint. The MA of the tibia was defined as a line at the midpoint of the tibia at the level of the knee joint to the centre of the tibial plafond at the ankle. The lateral distal femoral angle (LDFA) was defined as the lateral angle subtended by the MA of the femur and a line drawn across the articular surface of the distal femur at the most distal points of the lateral and medial femoral condyles. Similarly, the medial proximal tibial angle (MPTA) was defined as the angle subtended medially by the MA of the tibia and a line drawn between the most distal articular contours (Fig. 2).

### Computed tomographic and robotic analysis for CT-aHKA

**Fig. 3:**
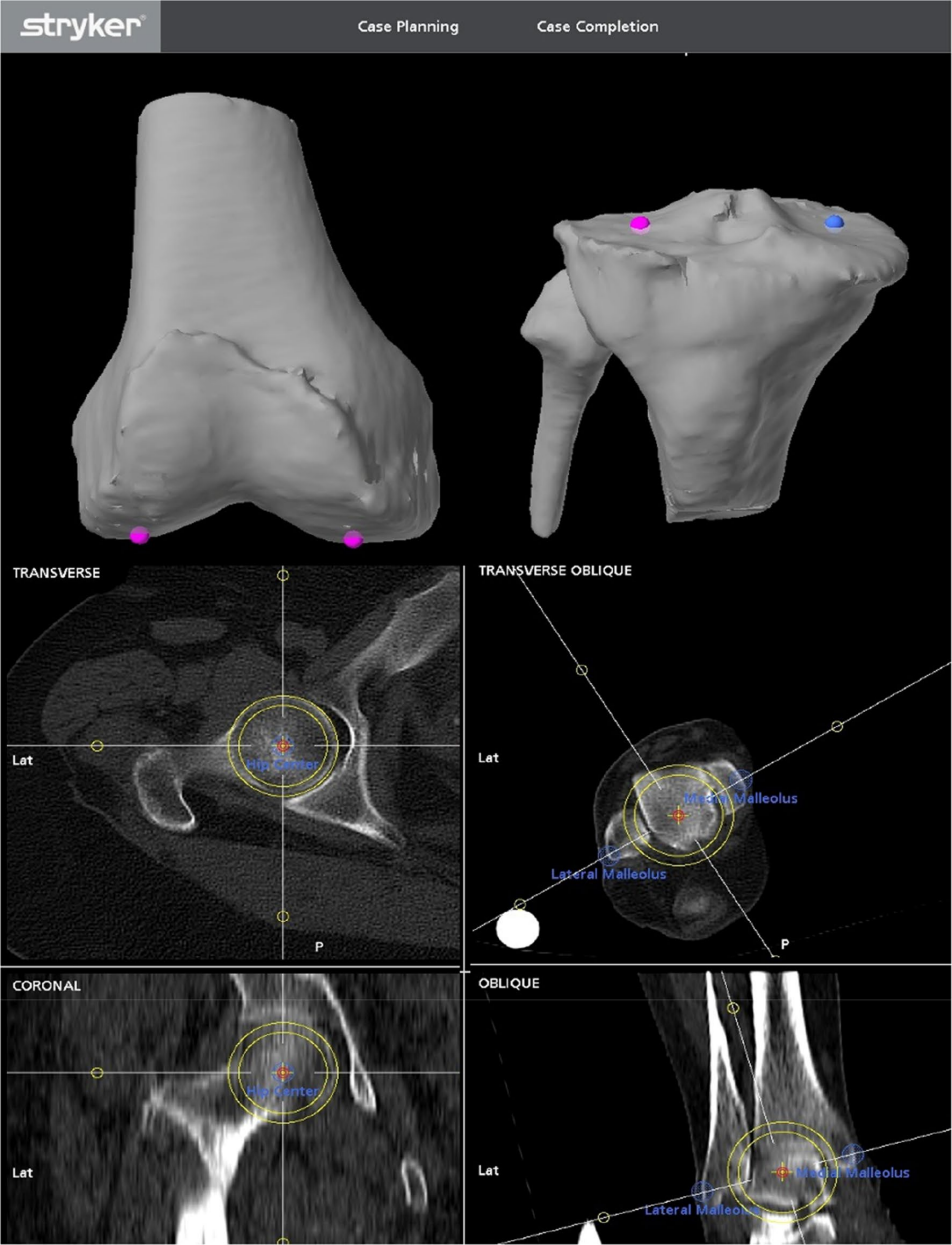
Mako computed tomographic robotic planning of alignment landmarks. CT images and 3D reconstructions were based on preoperative CTs within the Mako software, demonstrating distal femoral and proximal tibial landmarks and selection of the femoral head and ankle centre. Landmark acquisition was based on points at the most distal part of the condyles for the femur, and centre at 2/3 of the distance from the front of each plateau for the tibia, as demonstrated in the uppermost image

The same definitions were applied to determine landmarks using cross-sectional CT imaging and 3D reconstructions within the Mako software (Fig. 3).

**Fig. 4:**
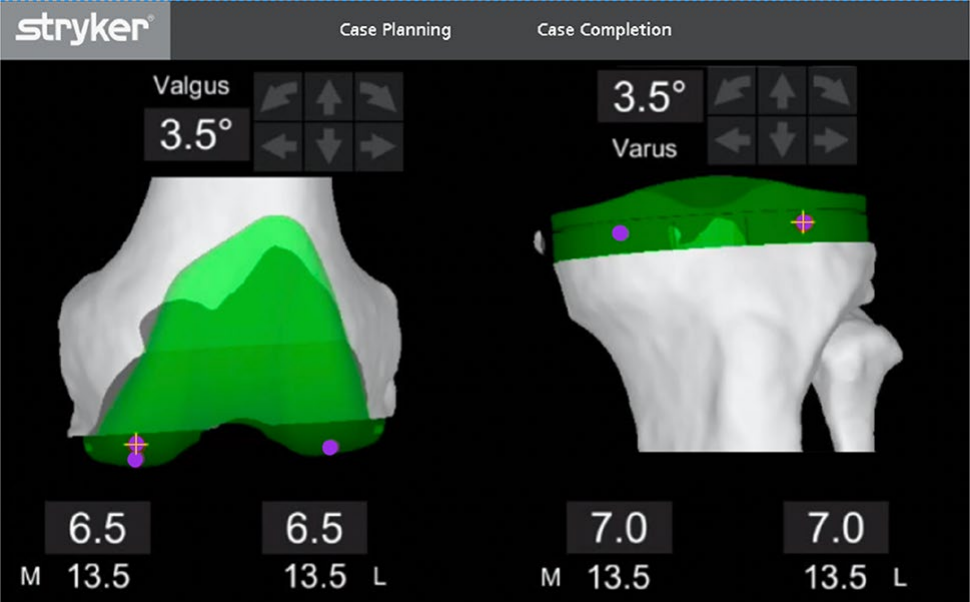
Computed tomographic determination of the arithmetic HKA angle. Determination of the computed tomographic arithmetic hip-knee-ankle angle (CT-aHKA) using the Mako software to determine the LDFA and MPTA. These are ascertained by application of matched resections (6.5 mm femur and 7.0 mm tibia) to the landmarks represented by the purple dots in this figure. The resultant coronal plane angle reported by the software (in this case 3.5° valgus for the femur and 3.5° varus for the tibia) is the precise deviation from the orthogonal axis. From these angles, we can infer the values of 86.5° for both the LDFA and MPTA and thereby calculate the CT-aHKA using the arithmetic method (CT-aHKA = MPTA − LDFA). *CT* computed tomographic, *aHKA* arithmetic hip-knee-ankle angle algorithm, *LDFA* lateral distal femoral angle, *MPTA* medial proximal tibial angle

The LDFA and MPTA in the CT group were calculated by application of equal-resection thicknesses to the distal femur and proximal tibia landmarks in the Mako planning screen (Fig. 4) as per a recently described technique on robotic KA planning by Clark et al. [12]. The angular deviation from neutral was used to estimate these two angles. For example, a valgus cut angle of 3.5° equates to an LDFA of 86.5°. Measurements from radiographs were carried out for all patients by the senior author and repeated independently by the orthopaedic fellow, with the mean of the two measurements used for analysis.

### Intraoperative technique for stressed HKA (sHKA)

**Fig. 5:**
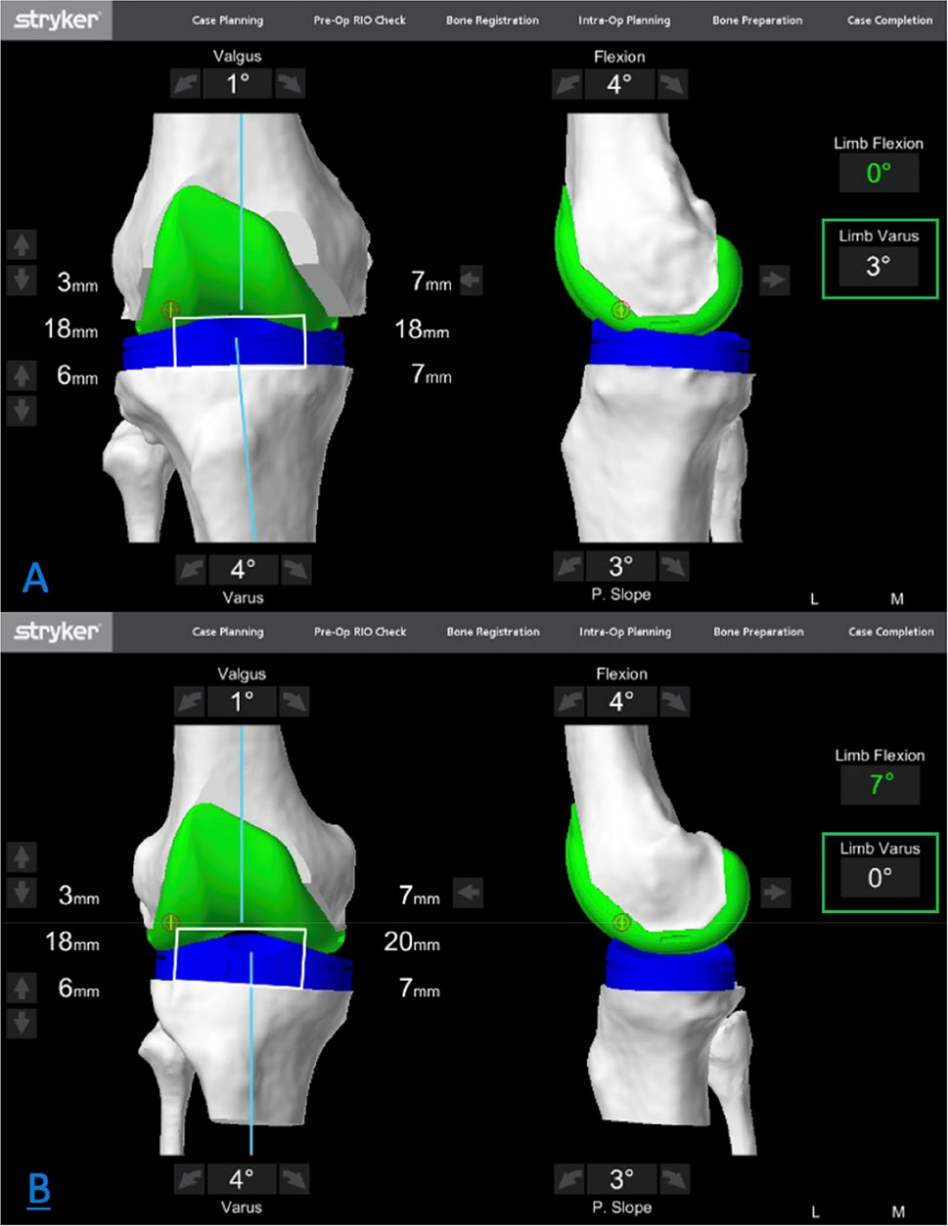
Intraoperative determination of the stressed hip-knee-ankle angle (sHKA). **A** Resting HKA of 3° varus (green square); **B** application of valgus stress to the same knee (varus deformity with loss of medial joint space), whilst in 7° of flexion, to determine the stressed HKA—in this case, an HKA of 0° (green square). The intended position of the implants is superimposed by the MAKO software to inform surgical workflow. *HKA* hip-knee-ankle angle

All patients underwent a medial parapatellar approach with excision of the anterior cruciate ligament and release of the deep medial capsular ligaments only. Following anatomical landmark registration [51], accessible osteophytes were removed and the arthrotomy was approximated using towel clips. The joint was then placed into extension, and the preliminary value for the HKA was recorded as the “resting HKA”. The knee was then stressed to reverse the direction of deformity, applying a valgus stress to the medial ligaments in knees with medial compartment osteoarthritis and a varus stress to the lateral ligaments in knees with lateral compartment osteoarthritis. The direction of applied force was the basis for inclusion into subgroups of medial and lateral compartment arthritis. This sHKA manoeuvre was performed with the joint flexed between 5° and 10° to de-tension the posterior capsule, which may act as a secondary restraint to deformity correction, particularly in the presence of significant posterior osteophytes. The HKA angle from the robotic navigation software was then recorded as the sHKA. Figure 5 shows an example of the robotic user interface during measurements for the resting and stressed HKA.

#### Outcomes

The primary outcome of this study was to determine the mean angular difference when the following groups were compared:

1. sHKA and aHKA
2. sHKA and CT-aHKA
3. aHKA and CT-aHKA
4. aHKA, CT-aHKA and sHKA (across all three groups)

Secondary outcomes were to determine if there was agreement in measurements between pairs 1 and 3 above. Further, comparison of sHKA relationship to the aHKA in patients with medial and lateral compartment arthritis was performed.

### Sample size calculation

In keeping with previous investigations [18, 33], a mean difference of 1.5° or less between measurements was considered indicative of equivalence and within the margin of error both for radiographic assessment and accuracy of optical computer-assisted navigation [26, 47]. Assuming the standard deviation (SD) of the paired differences to be 3.5°, to achieve a power of 80% at a 5% level of significance (two-sided), it was determined a minimum of 45 pairs would be required to detect whether a true difference exists between measurement techniques.

### Statistical analysis

Descriptive statistics were used for calculation of means, SD and 95% confidence intervals. Paired samples t-tests were used to compare means between any two groups, and repeated-measures ANOVA (rANOVA) was used for comparison of means across the three groups [36]. Bland–Altman plots were used to assess agreement between paired measurements [7]. These graphically represent the 95% limits of agreement (LOA) estimated by a mean difference ± 1.96 SDs within which measurements by the two methods were expected to lie [8]. For non-parametric comparisons in subgroups, the Wilcoxon signed-rank test was used. Tests for normality of distribution were conducted with Shapiro–Wilk test and Q-Q plots, and Mauchly's Test of Sphericity to determine if any corrections were required for rANOVA. Intraclass correlation coefficients (ICC) were used to assess inter-observer and intra-observer agreement in a subgroup of 15 patients, using a two-way mixed-effects model with absolute agreement. Statistical analysis was performed using SPSS 27 (IBM Corp, Armonk, NY, USA). Significance was set at a *p* value < .05.

## Results

Table 1 summarises demographic characteristics, and Table 2 describes the radiological parameters. On average, the resting HKA was approximately 3° more varus than the values for aHKA, sHKA, and CT-aHKA.

**Table 1:** Patient demographics

Variable	Value
Mean age, years (range)	68 (42–87)
Sex ratio, *n* (male:female)	41:35
Laterality, *n* (right:left)	37:51
Predominant arthritic compartment (medial:lateral)	73:15

n, number

**Table 2:** Radiological parameters

Variable	Mean ± SD (°)	Range (°)
mHKA	− 3.6 ± 6.7	− 16 to 19
rHKA	− 3.5 ± 4.5	− 14 to 12
aHKA	− 0.3 ± 3.9	− 9.1 to 12
CT-aHKA	− 0.5 ± 4.1	− 9 to 12
sHKA	− 0.4 ± 2.9	− 8 to 5

*SD* standard deviation, *mHKA* mechanical hip-knee-ankle angle, *rHKA* resting hip-knee-ankle angle, *aHKA* radiographic arithmetic hip-knee-ankle angle, *CT-aHKA* computed tomographic arithmetic hip-knee-ankle angle, *sHKA* stressed hip-knee-ankle angle

### Inter- and intra-observer agreement

Inter-observer agreement was rated as excellent, with an ICC of 0.94 (*p* < .001). Intra-observer agreement measured at a one-week interval was also excellent, with an ICC of 0.95 (*p* <.001).

### Primary outcome

Table 3 summarises the results for the primary outcome. There were no statistically significant differences between any of the paired comparisons, or across the three groups,

**Table 3:** Comparison of differences between aHKA, CT-aHKA, and stressed HKA

Compared variables	Mean ± SD (°)	95% CI (°)	*p* value
aHKA vs. sHKA	0.1 ± 4.2	− 0.8 to 1.1	n.s.*
aHKA vs. CT-aHKA	0.3 ± 5.3	− 0.8 to 1.4	n.s.*
CT-aHKA vs. sHKA	0.2 ± 4.1	− 0.7 to 1.0	n.s.*
aHKA vs. sHKA vs. CT-aHKA	N/A	N/A	n.s.^†^

*SD* standard deviation, *CI* confidence interval, *aHKA* radiographic arithmetic hip-knee-ankle angle, *sHKA* stressed hip-knee-ankle angle, *CT-aHKA* computed tomographic arithmetic hip-knee-ankle angle, *N/A* not applicable, *n.s.* not significant

*Paired samples *t* test, ^†^repeated measures ANOVA with Greenhouse–Geisser correction for asphericity

### Secondary outcome: agreement between groups

**Fig. 6:**
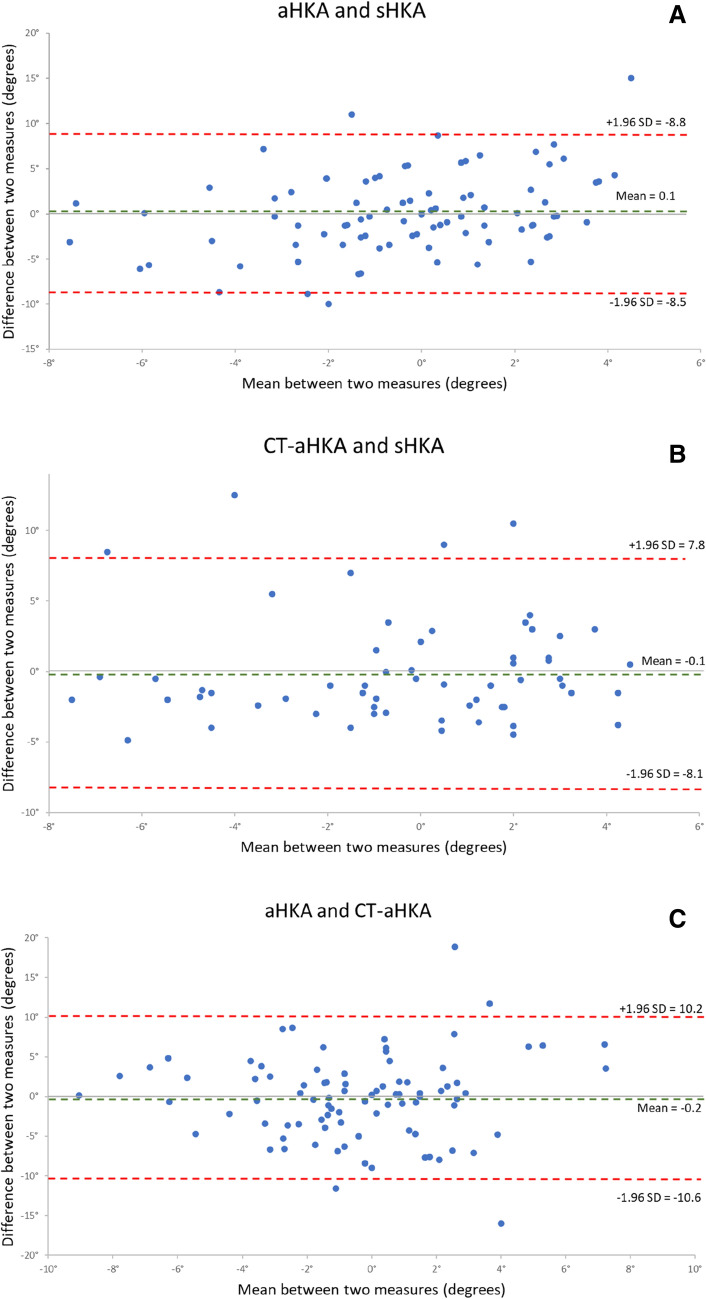
**A** Bland-Altman plot comparing the radiographic arithmetic hip-knee-ankle angle (aHKA) and the stressed hip-knee-ankle angle (sHKA). **B** Bland-Altman plot comparing the computed tomographic arithmetic hip-knee-ankle angle (CT-aHKA) and the stressed hip-knee-ankle angle (sHKA). **C** Bland-Altman plot comparing radiographic arithmetic hip-knee-ankle angle (aHKA) and the computed tomographic arithmetic hip-knee-ankle angle (CT-aHKA)

Figure 6 illustrates Bland-Altman plots for the compared variables. For aHKA versus sHKA, 83 out of 88 values (94.4%) fell within the LOA. For both the CT-aHKA versus sHKA and the aHKA versus CT-aHKA, 84 out of 88 (95.5%) fell within the LOA, which is consistent with good agreement [7, 40].

### Secondary outcome: compartmental arthritis correction patterns

Medial and lateral compartment arthritic correction patterns were analysed as subgroups, with the findings summarised in Table 4. Analysis of the lateral arthritis group showed a statistically significant difference (*p* <.001) in values between aHKA vs. sHKA, and CT-aHKA vs. sHKA. The medial arthritis group (73 knees) showed no differences in all three paired comparisons.

**Table 4:** Comparison of differences between aHKA, CT-aHKA, and stressed HKA

Compared variables	Medial compartment OA (mean ± SD) (°)	*p* value	Lateral compartment OA (mean ± SD) (°)	*p* value
aHKA vs. sHKA	− 0.1 ± 3.1	n.s.*	5.6 ± 3.3	.001^‡^
aHKA vs. CT-aHKA	0.3 ± 3.6	n.s.*	0.7 ± 3.2	n.s.^‡^
CT-aHKA vs. sHKA	0.5 ± 3.8	n.s.*	4.9 ± 3.3	.001^‡^

*aHKA* radiographic arithmetic hip-knee-ankle angle, *sHKA* stressed hip-knee-ankle angle, *CT-aHKA* computed tomographic arithmetic hip-knee-ankle angle, *SD* standard deviation, *n.s.* not significant

^‡^Wilcoxon signed-ranks test, *paired samples *t* test

## Discussion

The most important findings of this study were that there were no significant differences in the aHKA, sHKA, and CT-aHKA, thereby lending support to the use of the sHKA as an intraoperative validation of the aHKA to restore constitutional lower limb alignment in kinematically aligned TKA. Moreover, the findings establish the arithmetic HKA as an essential calculation for determining constitutional alignment prior to surgery using either radiographic or CT-based imaging.

A primary goal of KA is to restore constitutional limb alignment to minimise the need for intraoperative soft tissue releases and to provide more natural kinematics [6, 24, 41, 54]. It is therefore imperative that methods to determine the constitutional alignment are precise, reliable, and uncomplicated. The arithmetic HKA has been previously shown to closely approximate the constitutional alignment [33]. Our group uses it routinely to determine individualised alignment target plans preoperatively, and then uses the stressed HKA as a secondary intraoperative measure to validate the aHKA. We are unaware of any other study that has correlated these two methods to achieve this important target for surgeons who want to restore each patient’s pre-arthritic hip knee ankle angle in TKA surgery.

The concept of using “stressed” navigation, or radiographs, to determine if ligaments have become contracted is becoming increasingly discredited. Prior studies using stressed analyses (whether with radiographs or computer-assisted navigation) have aimed to restore the HKA angle to neutral, as mechanical alignment has been considered biomechanically advantageous [5, 13, 14, 19, 27]. An unintended and false extension of this assumption was that MA was also the “normal” alignment for every patient. However, an awareness of the wide variabilities in constitutional alignments, particularly over the last decade, has refuted this belief [4, 21]. Correcting the HKA to neutral will iatrogenically tighten ligaments on the same side as the constitutional deviation from neutral unless the constitutional alignment started in neutral. With only 15% of healthy and arthritic knees having an aHKA within ± 2° of neutral, ligament releases will be needed in a significant amount of MA TKAs [32, 34]. Further refuting the concept of ligaments contracting is the work of McAuliff et al. [37] and Okoamoto et al. [42] who have found that at least in varus knees, medial soft tissues do not contract, even with deformities of up to 15° of varus. Hence, the stressed HKA will tension the collateral ligamentous restraints on the side of osteochondral loss, providing a HKA angle target that reverses the arthritic deformity.

The use of a navigation-based “stress” pose and its implications for final alignment have been described by McEwen et al. to define constitutional alignment in KA, but not validated against the aHKA [38, 39]. In a recent paper, Sappey-Marinier et al. compared preoperative valgus stress radiograph in 749 patients undergoing primary TKA for varus arthritis to the aHKA [46]. They found that the valgus correction angle was similar to the aHKA, confirming its utility in approximating the constitutional alignment. The findings in the current study therefore corroborate theirs, although in a smaller sample. There are, however, several relevant differences. First, our method relies on intraoperative optical tracking, which is considered more precise [15] and is free of radiation exposure risk to either the patient or the operator. Second, stressed view acquisition is part of the routine surgical process of primary robotic TKA with minimal additional time, cost implications, or disruptions to the workflow. Finally, the study by Sappey-Marnier et al. only compared values between stressed radiographs and radiographic aHKA measurements only.

The use of preoperative CT imaging is becoming a routine assessment in some robotic-assisted TKA platforms. Using imaging data from these surgeries, the aHKA measurements using CT also showed a high level of agreement with the routine method of radiographic assessment. Both methods also showed similar concordance with the sHKA. Whilst this does not abrogate the use of CT in the setting of robotic-assisted KA, the similarity of the measurements between the two methods does provide further validation of the aHKA, whether derived from radiographs or from CT imaging.

Regarding the subgroup analysis of 15 knees with lateral compartment arthritis (varus correction for sHKA), no significant difference was found only when comparing the two groups that had measurements taken using the arithmetic method (aHKA and CT-aHKA), but not when these groups were compared to the stressed HKA. In contrast, the medial compartment group showed no difference across all group comparisons. This finding is not unexpected, as knees with lateral compartment arthritis are likely to behave differently under applied varus loads due to the high degrees of variability in constitutional lateral laxities compared to medial laxities [2, 17, 35]. Medial ligamentous structures, in both flexion and extension, typically exhibit less coronal plane laxity than lateral sided structures [45, 48, 53]. Therefore, it is likely that the stressed HKA is more reliable in knees with medial arthritis. However, it should also be considered that the numbers in the lateral compartment arthritis cohort were small.

Our study has several limitations. The first pertains to the arithmetic method, as it does not consider the small contribution of the joint line convergence angle (0.5°), nor does it compensate for bone loss in the presence of severe arthritis [18]. Second, the sHKA may not be reliable in knees where significant joint space loss in both medial and lateral compartments is present. Third, using the stressed HKA to plan alignment is a technique that is only possible in computer-assisted or robotic-assisted TKA, a technology that may have significant barriers to implementation, although arguably, these barriers are becoming less challenging [1, 9, 49]. Hence, it might be reasonable to consider the aHKA as a tool to provide an initial alignment target, with the sHKA acting to validate the plan with either computer-assisted or robotic-assisted technologies.

Despite these limitations, these findings provide greater certainty for surgeons when using the aHKA and sHKA for pre-operative planning with intra-operative restoration of constitutional alignment when undertaking KA TKA.

## Conclusion

This study found that the values obtained for the arithmetic HKA and stressed HKA, whether obtained using CT or radiography, are not significantly different. These findings therefore validate the arithmetic method to determine constitutional alignment and support the stressed HKA as a technique to approximate constitutional alignment intraoperatively.
